# Immunization with Multiple Virulence Factors Provides Maternal and Neonatal Protection against Group B Streptococcus Serotypes

**DOI:** 10.3390/vaccines11091459

**Published:** 2023-09-05

**Authors:** Jie Wang, Wenbo Li, Ning Li, Beinan Wang

**Affiliations:** 1 Key Laboratory of Pathogenic Microbiology and Immunology, Institute of Microbiology, Chinese Academy of Sciences, Beijing 100101, China; 2 Beijing Varnotech Biopharm Ltd., Beijing 100176, China

**Keywords:** group B streptococcus, virulence factors, intranasal immunization, vaginal colonization, maternal vaccines

## Abstract

Group B streptococcus (GBS) commonly colonizes the vaginal tract and is a leading cause of life-threatening neonatal infections and adverse pregnancy outcomes. No effective vaccine is clinically available. Conserved bacterial virulence factors, including those of GBS, have been employed as vaccine components. We investigated serotype-independent protection against GBS by intranasal immunization with six conserved GBS virulence factors (GBSV6). GBSV6 induced systemic and vaginal antibodies and T cell responses in mice. The immunity reduced mouse mortality and vaginal colonization by various GBS serotypes and protected newborn mice of immunized dams against GBS challenge. Intranasal GBSV6 immunization also provided long-lasting protective immunity and had advantages over intramuscular GBSV6 immunization regarding restricting vaginal GBS colonization. Our findings indicate that intranasal immunization targeting multiple conserved GBS virulence factors induces serotype-independent immunity, which protects against GBS infection systemically and vaginally in dams and prevents newborn death. The study presents valuable strategies for GBS vaccine development.

## 1. Introduction

*Streptococcus agalactiae*, or group B streptococcus (GBS), is an encapsulated Gram-positive opportunistic pathogen that can be classified into 10 different serotypes (Ia, Ib, II–IX) based on the antigenic and structural properties of the capsular polysaccharide (CPS). GBS is found in the vagina or lower gastrointestinal tract of about 10–40% of women [1] and can cause chorioamnionitis, preterm birth, and stillbirth [2,3], as well as neonatal pneumonia, sepsis, and meningitis with early-onset disease (EOD) or late-onset disease (LOD) due to ascending infection or transmission during delivery [4]. GBS is also responsible for morbidity and mortality in gravidas, the elderly, and immunocompromised adults. The only available treatment, intrapartum antibiotic prophylaxis (IAP), has substantially reduced the incidence of EOD. However, in some developing countries where IAP has not been implemented, EOD incidence is still a leading cause of neonatal mortality. Additionally, LOD, preterm birth, and stillbirth are not preventable by IAP, remaining a public health problem in both high- and low/middle-income countries [5]. Hence, improved preventive measures are urgently needed to fully address the burden of GBS disease for gravidas and infants.

Maternal vaccines, which lead to placental transfer of GBS antibodies, protect against multiple outcomes of GBS infection [6]. Currently, there are no approved GBS vaccines available. Two major types of GBS vaccine have been developed: CPS–protein conjugate vaccines and protein-based vaccines. CPS–protein conjugate vaccines have shown the most promising results to date in clinical studies [4]. However, there are some concerns regarding serotype replacement/capsular switching, lack of coverage of non-typeable isolates, and reduced efficacy owing to the diverse serotype distribution across and within geographic regions [7]. Protein-based vaccines may confer broad protection across all GBS serotypes and may also alleviate concerns regarding the threat of serotype replacement/capsular switching [4,8,9]. Several research groups have identified various surface-exposed proteins of a wide panel of clinical GBS strains and serotypes that are able to induce opsonically active antibodies, such as recombinant alpha-like protein subunit vaccines (GBS-NN and AlpN) that are currently under clinical investigation as broad-spectrum vaccines [10,11]. 

The selection of optimal bacterial antigens is crucial for vaccine efficacy. Promising antigens are: (1) surface-exposed or secreted in order for them to be more accessible to antibodies, (2) conserved across a wide range of serotypes so that they can confer cross-serotype protection, (3) highly immunogenic in order for them to induce T and B cell responses, and (4) able to induce long-lasting protective immunity [12,13]. There is increased awareness of the involvement of GBS protein antigens not only in immunity but also in pathogenicity [14]. Clinical GBS strains have evolved a wide variety of virulence factors [15], including adhesins that mediate GBS colonization of the vaginal tract, enabling transmission to newborns to cause EOD [16]. Other virulence factors, such as FbsB and ScpB, can facilitate GBS invasion of various tissues, damaging the tissue structure, which leads to dysfunction of human organs [17,18]. In gaining access to the blood circulation, Srr2 can help GBS to invade the brain epithelium, causing meningitis [19]. These virulence factors induce protective immunity in mouse models of infection when used individually as antigens. 

However, single-antigen vaccines may provide variable protection in preclinical models [20], as immunity induced by a single virulence factor might be ineffective against isolates lacking the target antigen or inadequate due to the presence of other crucial virulence factors [21]. Thus, including multiple virulence factors involved in various GBS pathogenic mechanisms in a vaccine would improve protection against GBS diseases after induction of antibodies. These antibodies would not only facilitate bacterial clearance via opsonophagocytosis but would also neutralize the pathogenic functions of the virulence factors during an infection.

In this study, a combination of six GBS virulence factors involved in different steps of GBS pathogenesis was used as a vaccine (GBSV6) and delivered through the intranasal (i.n.) route. The six conserved GBS proteins were: (1) sortase A (SrtA), which is necessary for anchoring cell wall proteins (including many virulence factors) [22]; (2) cell-surface-associated protein A (CspA), which cleaves CXC chemokines [23]; (3) C5a peptidase (ScpB), which specifically cleaves complement-activated C5a, abolishing neutrophil recruitment to the infection site [24]; (4) a secreted fibrinogen-binding protein (FbsB), which enhances GBS internalization into epithelial cells [25]; (5) fibrinogen-binding protein with serine-rich repeat 2 (Srr2), which increases the permeability of the blood–brain barrier [26], and (6) an immunogenic bacterial adhesin (BibA), which specifically binds to C4-binding protein to resist opsonophagocytic killing by neutrophils [21,27]. These proteins have been reported to elicit protective antibodies [28,29,30,31] or serotype-independent protection [32,33]. Vaginal mucosal immunity is necessary for efficient local GBS clearance [34]. Mucosal immunization through the i.n. route not only stimulates an immune response in the respiratory tract, but also induces a strong genital/vaginal mucosal immune response [35,36]. We hypothesized that i.n. immunization with the set of six conserved virulence factors would ensure the efficacy and coverage of the GBS vaccine. In this study, we showed that i.n. GBSV6 immunization of mice reduced GBS colonization of the vagina, protected against lethal infections, and increased the survival of newborn mice at risk of infection with various serotypes.

## 2. Materials and Methods

### 2.1. Ethics Statement

This study was performed in strict accordance with the recommendations in the Guide for the Care and Use of Laboratory Animals of the Institute of Microbiology, Chinese Academy of Sciences (IMCAS) Ethics Committee. The protocols were approved by the Committee on the Ethics of Animal Experiments of IMCAS (permit number: SQIMCAS20211128). All animal experiments, including vaccination, inoculation, and sample collection, were conducted under isoflurane anesthesia, and all efforts were made to minimize the suffering of the animals. The collection and use of human serum samples were approved by the Ethics Committee of Beijing Children’s Hospital (2018-162). Informed consent was obtained from the human subjects prior to enrollment in the study.

### 2.2. Bacterial Strains and Culture Conditions

GBS serotypes Ib, III (a), and III (b) were clinical isolates obtained from Beijing Children’s Hospital. All strains were grown in Todd-Hewitt broth (THB; BD Bioscience, San Jose, CA, USA) or blood agar plates at 37 °C in 5% CO_2_. Overnight cultures were pelleted, washed, and resuspended in PBS and then used for animal challenge studies. CFUs were verified by plating on blood agar plates.

### 2.3. Cloning and Expression of Recombinant Proteins

The DNA sequences of SrtA and BibA were amplified from GBS serotype Ib. The DNA sequences of Srr2, FbsB, ScpB, and CspA were amplified from GBS serotype III (a). The primers used for the amplification are listed in Table S1. Recombinant SrtA (amino acids 82 to 247) and Srr2 (amino acids 192 to 543) were cloned into the pET28a vector as previously described [19,37]. Recombinant FbsB (amino acids 19 to 628) and BibA (amino acids 34 to 471) were cloned into the pCold-SUMO vector [16,18]. For CspA and ScpB, mutations were generated to remove their toxic enzymatic activity. ScpB (amino acids 32 to 1032), with D130A and S512A site mutations, was cloned into the pET-28a vector [17]. CspA (amino acids 36 to 1076), with D172A and S567A site mutations, was cloned into the pET-28a vector [29]. DNA sequences of ScpB and CspA were synthesized by Shanghai Generay Biotech Co., Ltd. All constructs were transformed into *Escherichia coli* BL21 (DE3) for expression. Recombinant proteins were purified as previously described [18]. Lipopolysaccharide was removed from the purified proteins to <0.1 endotoxin units (EU)/μg recombinant protein, using a ToxinEraser endotoxin removal kit (Genscript, USA), following the manufacturer’s protocol.

### 2.4. Sample Collection and Preparation

The blood samples were collected through the tail vein of mice and the serum was obtained by centrifugation, and then stored at −20 °C for subsequent analysis. GBS loads in vaginal lumen were quantified post-challenge, as previously described [34]. Briefly, a pre-wetted swab was carefully inserted into the vaginal lumen and gently rotated. The swabs were soaked and serially diluted in PBS with 0.1% bovine serum albumin and protease inhibitors for CFU counting on plates. Samples of reproductive tract tissue, bone marrow, and spleen cells were collected as described by Patras et al. [38]. Briefly, mice were euthanized by exposure to carbon dioxide gas in a rising concentration. The female reproductive tract (FRT) tissue was taken, diced into small pieces, and digested with collagenase IV (Roche Life Science, Indianapolis, IN, USA) for 30 min. Then, the FRT samples were strained to remove larger clumps before laying on Percoll gradients for lymphocyte isolation. The collected cells were filtered and washed to generate single-cell suspension. The spleen and bone marrow (BM) were removed and dissociated into single-cell suspensions in complete RPMI-1640 medium. The preparation was centrifuged, and the pelleted cells were analyzed by ELISpot assays for antibody-producing B cells and IL-17A-producing T cells. All the tissues were treated with ammonium-chloride-potassium lysis buffer to lyse the red blood cells.

### 2.5. Enzyme-Linked Immunosorbent Assay (ELISA)

Antigen-specific antibodies in mouse and human sera were measured by standardized ELISA. Human serum samples were collected from 96 child patients (aged 5–15 years). The patients had respiratory infections due to various identified or non-identified pathogens. In brief, Corning Costar 96-well plates (Fisher Scientific, Waltham, MA, USA) were coated with 5 μg/mL of GBSV6 or each of the six proteins at 4 °C overnight. For bacteria coating, surface protein extraction of GBS was prepared using the protocol described by Hughes et al. [39]. Then, 100 μL/well of the extraction was added to coat the plates. After antigen removal, the plates were incubated with sealing buffer (200 μL/well of 5% skim milk) at 37 °C for 2 h. Next, 100 μL of each of the 1:1000 diluted mouse or human serum samples were added to the plates following washing with PBS buffer three times. After 2 h of incubation at 37 °C, the serum samples were removed, and the wells were rinsed with PBST buffer six times. Then, 1:4000 diluted HRP-conjugated goat anti-mouse IgG, or goat anti-mouse IgA antibody (Southern Biotech, Birmingham, AL, USA), was added. Following 1 h of incubation at 37 °C and washing with PBST buffer six times, TMB Substrate (Tiangen Biotech, Beijing, China) was added (50 μL/well) and incubated for 15–30 min at 37 °C under dark conditions. Stop Solution (50 μL/well) was added to stop the reaction. The ELx800 plate reader (BioTek, Winooski, VT, USA) was used to measure absorbance at 450 nm, and the absorbance at 630 nm was used as the internal control. A standard curve was generated by adding double-diluted purified mouse IgG (Alpha Diagnostic Int. Inc., San Antonio, TX, USA) or IgA (Bethyl Laboratories, Montgomery, TX, USA) to anti-mouse IgG- or anti-mouse IgA-coated wells. The concentration of the antibody levels was calculated according to the standard curve.

### 2.6. Enzyme-Linked Immunospot (ELISpot) Assays

ELISpot assays of T and B cells were conducted as previously described [40]. Antigen-specific IL-17A-secreting cells or IFN-γ-secreting cells were detected with kits of Mouse IL-17A ELISPOT BASIC or Mouse IFN-γ ELISPOT BASIC (Mabtech, Minneapolis, MN, USA) according to the manufacturer’s instructions. Briefly, the PVDF membranes were pretreated with 35% ethanol, and the plates were coated with mouse anti-IL-17-I or AN18 for 24 h. Single-cell suspensions of the FRTs, bone marrow, or spleen cells (4 × 10^5^) were added with or without 2.0 μg/100 ul of PBS of GBSV6, or 2.0 ug each of the six proteins of GBSV6 in 100 ul of PBS overnight. Plates were washed and incubated with 100 μL of 0.25 μg/mL of biotinylated monoclonal antibody MT2270 or 1 μg/mL of R4-6A2, followed by Streptavidin–HRP. The plates were developed using established procedures by 3-amino-9-ethylcarbazole (AEC) (Millipore, Bedford, MA, USA). Spots were enumerated with an ImmunoSpot Analyzer (Cellular Technology Ltd., Beachwood, OH, USA) and multiplied by the dilution factor.

### 2.7. GBS Opsonophagocytic Assay

The opsonophagocytic killing assay (OPKA) was conducted with HL-60 cells as described previously [41], with modifications. The HL-60 cell line is a promyelocytic cell line derived from human leukemia. The cells can be induced to differentiate into granulocyte-like cells, or neutrophils, and widely used for OPKA to measure the efficacy of bacterial vaccines. Briefly, HL-60 cells were differentiated into neutrophil-like cells by culturing in RPMI medium (Invitrogen, Life Technologies Ltd., CA, USA) containing 0.8% N, N-dimethylformamide (DMF) for 5–7 days. OPKA reactions were performed in 96-well plates with heat-inactivated (56 °C for 30 min) serum from immunized mice, 1 × 10^3^ CFU of GBS in opsonization buffer, 2 × 10^6^ differentiated HL-60 cells, and 10% baby rabbit complement (Pel-Freez, Rogers, AR, USA). Control reactions were performed with non-immune serum or without GBS cells. The mixtures were incubated on a mini-orbital shaker (700 rpm) for 45 min at 37 °C in a 5% CO_2_ atmosphere. The reaction mixture from each well was collected, serially diluted, and plated on blood agar plates. The experiment was performed in triplicate. CFUs were counted after leaving overnight at 37 °C in a 5% CO_2_ atmosphere. The percentage of killed bacteria was determined by comparing the CFUs in tests carried out without effector cells (100% surveillance) to those of the tested samples, subtracting the percent that survived from 100%.

### 2.8. GBS Adherence Assays

The adherence assay was performed as previously described [42]. A549 cells (human lung carcinoma cells, ATCC CCL185) were grown to confluency in 24-well plates overnight. GBS (3 × 10^6^) was mixed with anti-GBSV6 serum or serum from CpG control mice (1:1, *v*/*v*) and incubated for 30 min. Then, samples were added to A549 cells at a multiplicity of infection of 10 and incubated for 2 h. Cells were washed with PBS 6 times and 0.1% Triton X-100 was added to lyse the cells. Lysate was plated on selective blood agar plates to enumerate bacterial CFUs. The adherent CFU percentage was calculated as: [(recovered CFU)/(original inoculum CFU)] × 100%.

### 2.9. GBS Invasion Assay

The human brain microvascular endothelial cell line (hBMEC) was kindly provided by Prof. Xiang-Ru Wang from Huazhong Agricultural University, Wuhan, Hubei, China, and cultured as described by Yang et al. [43]. The bacterial invasion assay was performed using hBMEC, as previously described [44]. Briefly, GBS was grown overnight in THB and then added to confluent hBMEC monolayers at a multiplicity of infection of 0.1 (1 × 10^4^ CFU). Then, 30 min after infection, monolayers were treated with a penicillin and streptomycin mixture (100 μg/mL) for 30 min to remove extracellular GBS, and cells were lysed with 0.1% Triton X-100. Cell lysates were plated on blood agar plates to enumerate the intracellular bacteria. To determine the inhibitory effects of anti-GBSV6 serum on GBS invasion, 250 μL of GBS (2.5 × 10^5^ CFU) was preincubated with 250 μL of mouse immune serum for 30 min at room temperature, and 20 μL of GBS (1 × 10^4^ CFU) was added to the cells. GBS invasion was calculated as: [(recovered CFU)/(initial inoculum CFU)] × 100%.

### 2.10. Mice Immunization and Challenge

Specific pathogen-free (SPF) female CD-1 mice (6-week-old; Vital River Laboratory Animal Center, Beijing, China) were immunized (30 μL) 3 times (days 0, 7, and 14) through the intranasal (i.n.) or intramuscular (i.m.) route with 60 μg of GBSV6 (10 μg of each recombinant protein) and 10 μg of CpG-oligodeoxynucleotides (CpG-OND 1826; Generay Biotechnology, China) [45].

Vaginal colonization and lethal challenge experiments were performed with 10-week-old SPF female CD-1 mice. For vaginal colonization, a mouse model described by Patras et al. was employed [38]. In brief, mice were intraperitoneally injected with β-estradiol (0.5 mg/mouse) and vaginally challenged with GBS serotype Ib or GBS III (a) (1.0 × 10^7^/10 μL) 2 weeks after the last immunization. For systemic infection, dose escalation studies were conducted to determine the lethal challenge dose. Mice were challenged i.v. with 5 × 10^7^, 1 × 10^8^, and 2 × 10^8^/mouse, respectively. The survival rates were monitored, whereby 2 × 10^8^ of GBS serotype Ib or serotype III (a) caused 70–90% death within 10 days after the challenge and was used as a lethal challenge dose. Mice were challenged with the lethal dose of GBS serotype Ib or III (a) through the tail vein. Survival was monitored twice per day for 10 days. Mice with a weight loss of 25% of their starting body weight were euthanized and recorded as dead.

The maternal immunization/newborn challenge experiment was performed as described by Buccato et al. [46]. In brief, CD-1 female mice were mated 3 days after the last immunization. Within 48 h of birth, the newborns were intraperitoneally challenged with 2 × 10^6^ CFUs of GBS serotype Ib or III (which is lethal to 80–90% of newborns). Survival of the newborns was monitored for 3 days after challenge. Mortality was an anticipated outcome and was approved by the Animal Ethics Committee. Surviving mice were euthanized at the end of the experiment.

### 2.11. Statistical Analyses

Statistical analyses were performed in GraphPad Prism v7.0. Vaginal CFUs were compared using two-tailed unpaired Mann–Whitney U tests. Normally distributed continuous variables were compared between two groups using unpaired, two-tailed Student’s t tests. Normally distributed continuous variables were compared among more than two groups using one- or two-way analysis of variance, followed by Tukey’s multiple comparisons tests. The survival and percentage of colonized mice were compared over time between groups using log-rank (Mantel–Cox) tests. Survival of newborns was compared using Fisher’s exact test. Statistical significance was defined as *p* < 0.05.

## 3. Results

### 3.1. Immunization i.n. with GBSV6 Induced Antibody Responses in the Blood and Vagina

**Figure 1:**
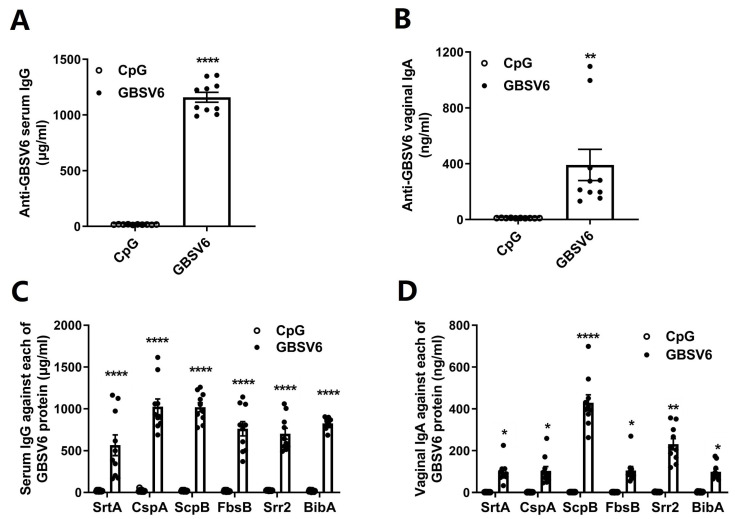
Intranasal (i.n.) GBSV6 immunization induced antibody responses in the blood and vagina. Mice were i.n. immunized 3 times at 1-week intervals with GBSV6 or CpG. Two weeks after the last immunization, GBSV6-specific antibodies were measured in (**A**) serum and (**B**) vaginal lavage fluid by the enzyme-linked immunosorbent assay (ELISA). (**C**) Serum IgG and (**D**) vaginal IgA targeting each of the GBSV6 antigens. Data represent mean ± standard error of the mean (SEM) based on two independent experiments (n = 10). * *p* < 0.05, ** *p* < 0.01, **** *p* < 0.0001.

The virulence factors, including SrtA, ScpB, CspA, FbsB, Srr2, and BibA, play various roles in GBS pathogenesis and are expressed by highly pathogenic strains, a variety of clinical isolates, or almost all GBS serotypes. The selected proteins were cloned, expressed, and purified, and their purity was determined by SDS-PAGE (Figure S1). They were then pooled (10 μg of each) and designated GBSV6. Mice were immunized with i.n. GBSV6, along with CpG-oligodeoxynucleotides (CpG) as an adjuvant, in phosphate-buffered saline (PBS) three times at one-week intervals. Control mice were inoculated with CpG in PBS. The enzyme-linked immunosorbent assay (ELISA) revealed that GBSV6-specific antibodies in the serum (IgG) and vaginal lavage fluid (IgA) were induced in the immunized mice (Figure 1A,B). These serum IgG and vaginal mucosal IgA were directed against each of the GBSV6 proteins (Figure 1C,D), with strong serum IgG responses to ScpB and CspA and strong vaginal IgA responses to ScpB. These results indicate that i.n. GBSV6 is highly immunogenic and induced strong antibody responses both systemically and locally.

### 3.2. GBSV6-Specific Antibodies Recognized Clinical GBS Isolates

**Figure 2:**
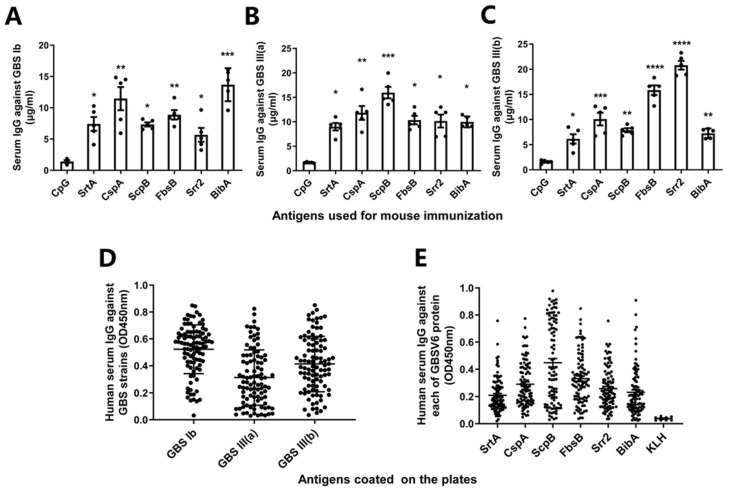
GBSV6-specific antibodies recognized GBS cells and were antigenic in humans. Serum IgG responses to GBS serotypes (**A**) Ib, (**B**) III (a), and (**C**) III (b) in mice immunized with each GBSV6 antigen, determined by the ELISA. Data represent mean ± SEM (n = 5). Human serum IgG responses to (**D**) various GBS serotypes and (**E**) to each of the GBSV6 antigens. Data represent mean ± SEM (n = 5). Keyhole limpet hemocyanin (KLH) was used as a negative control (n = 96). * *p* < 0.05, ** *p* < 0.01, *** *p* < 0.001, **** *p* < 0.0001.

GBSV6 contains surface proteins. We determined whether the GBSV6-specific antibodies could recognize clinical GBS isolates. Sera from mice i.n. immunized with each GBSV6 protein individually had higher levels of bacterial binding to GBS serotypes Ib, III (a), and III (b) compared to sera from CpG control mice, according to the ELISA (Figure 2A–C). This indicates that the antibodies induced in GBSV6-immunized mice can recognize native target molecules on the bacterial surface.

### 3.3. Antibody Response to GBSV6 in Humans

GBS is a frequent isolate from children. Serum samples from 96 child patients were collected. These patients had respiratory infections due to various identified or non-identified pathogens. The ELISA showed that the serum samples bound to clinical GBS serotypes Ib, III (a), and III (b) (Figure 2D). They also had higher mean responses to each GBSV6 protein compared to a non-related antigen (keyhole limpet hemocyanin, KLH) (Figure 2E). These results indicate that human antibodies specifically recognize GBS and GBSV6 antigens.

### 3.4. GBSV6 Induced T Cell Responses

**Figure 3:**
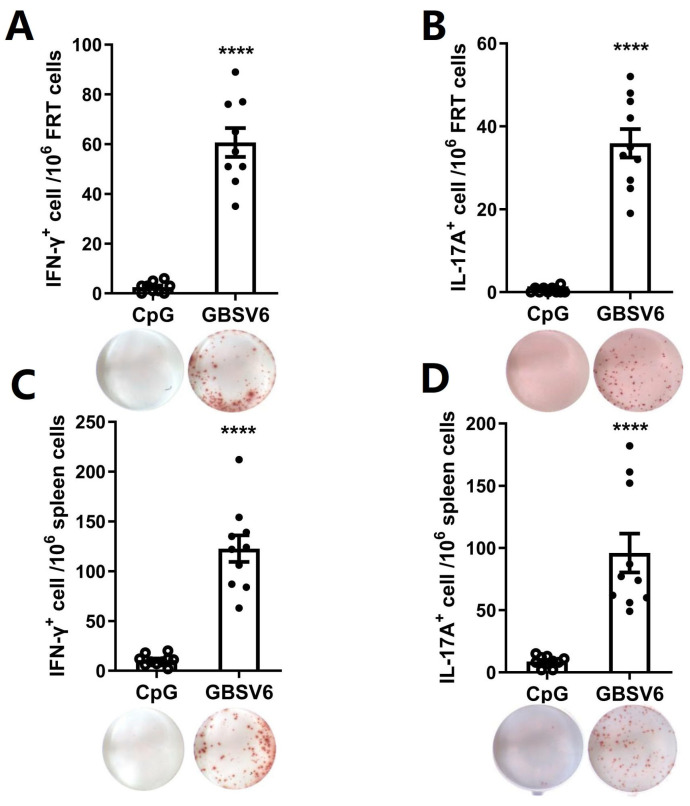
T cell responses to GBSV6. Mice were immunized as described in Figure 1. Two weeks after the last immunization, the FRT and spleen samples were collected. (**A**) IL-17A- and (**C**) IFN-γ-secreting T cells in the FRTs, and (**B**) IL-17A- and (**D**) IFN-γ-secreting T cells in the spleens, in response to GBSV6, were determined by ELISpot. Data represent mean ± SEM based on two independent experiments (n = 10). **** *p* < 0.0001.

The Th17 response to GBSV6 was determined in the female reproductive tracts (FRTs) and spleen by the T cell enzyme-linked immunospot assay (ELISpot) 2 weeks after the last immunization. High numbers of IL-17-secreting CD4+ T cells were found in the FRTs and spleens of GBSV6-immunized mice, while these cells were not detected in the CpG control mice (Figure 3A,B). Higher numbers of IFN-γ-secreting CD4+ T cells were also detected in GBSV6-immunized mice compared to CpG control mice (Figure 3C,D). These results indicate that i.n. GBSV6 immunization induces Th17 and Th1 responses in mucosal and systemic lymphoid tissues.

### 3.5. GBSV6-Specific Antibodies Promoted GBS Opsonophagocytosis and Prevented GBS Adherence to and Invasion of Host Cells

**Figure 4:**
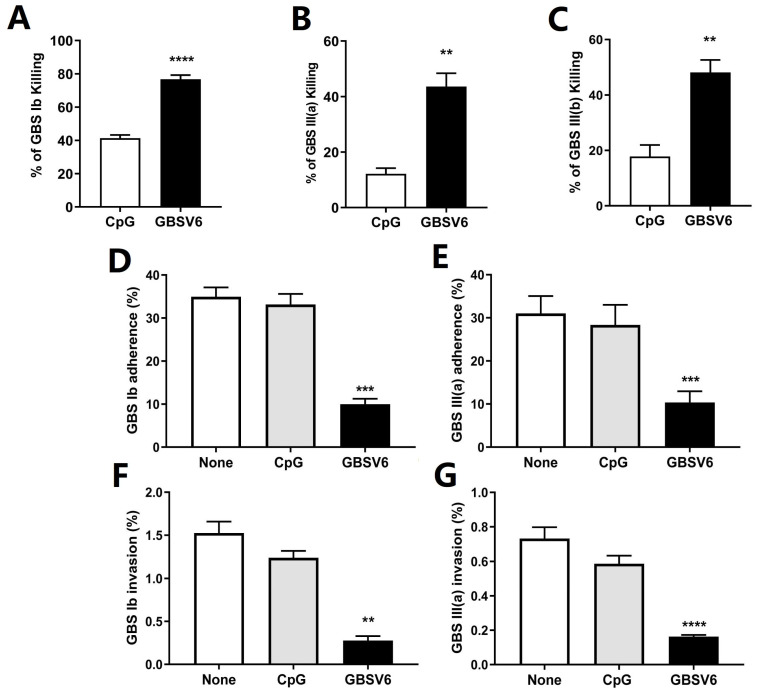
GBSV6-specific antibodies promoted GBS opsonophagocytosis and prevented GBS adherence to and invasion of host cells. GBS serotypes (**A**) Ib, (**B**) III (a), and (**C**) III (b) were incubated with differentiated HL-60 cells in the presence of sera from GBSV6-immunized or CpG control mice for 45 min, and then the bacterial growth rate was determined by CFU counting. For the adherence assay, A549 cells were infected with GBS serotype (**D**) Ib or (**E**) III (a) at a multiplicity of infection of 10 for 2 h, washed to remove the non-adhered bacteria, lysed, and plated onto blood agar plates for CFU counting. For the invasion assay, monolayers of human brain microvascular endothelial cells (hBMECs) were infected with GBS serotype (**F**) Ib or (**G**) III (a) for 30 min, washed and incubated with penicillin and streptomycin for 30 min to remove extracellular bacteria, lysed, and plated onto blood agar plates for CFU counting. Data represent mean ± SEM (n = 5). ** *p* < 0.01, *** *p* < 0.001, **** *p* < 0.0001.

More bacteria were killed when GBS strains were pretreated with sera from GBSV6-immunized mice than sera from CpG control mice, and the increased opsonophagocytic killing was observed for various GBS serotypes, i.e., Ib, III (a), and III (b) (Figure 4A–C).

The roles of GBSV6-specific antibodies in the prevention of GBS adherence to and invasion of host cells were evaluated using A549 cells (human lung epithelial cells) and human brain microvascular endothelial cells (hBMECs), respectively. GBS adherence to A549 cells (Figure 4D,E) or invasion of hBMECs (Figure 4F,G) were significantly reduced when GBS strains were pretreated with sera from GBSV6-immunized mice compared to sera from CpG control mice. These results indicate that GBSV6-specific antibodies can impede bacterial attachment to epithelial cells (preventing colonization) and inhibit bacterial penetration of the host brain endothelium (preventing crossing of the blood–brain barrier).

### 3.6. GBSV6 Immunization Promoted GBS Clearance in the Vagina and Protected against Systemic GBS Infection

**Figure 5:**
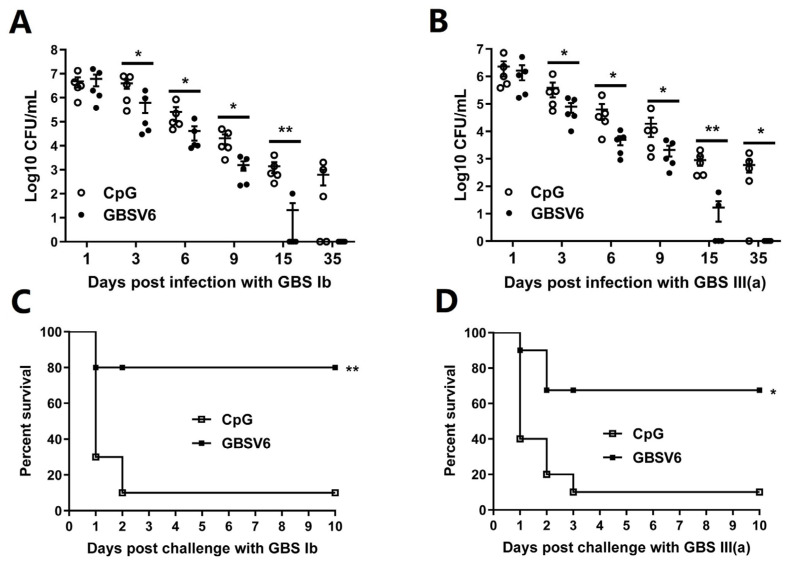
GBSV6 protected against vaginal and systemic infections by both GBS serotype Ib and III (a). Mice were immunized as described in Figure 1. Two weeks after the last immunization, the mice were intravaginally challenged with 1 × 10^7^ CFUs of GBS serotype (**A**) Ib or (**B**) III (a) and then CFUs in vaginal lavage fluid were counted at the indicated time points over the following 35 days. Data represent mean ± SEM (n = 5). Two weeks after the last immunization, the mice were administered a lethal dose of GBS serotype (**C**) Ib or (**D**) III (a) through the tail vein, and the mortality of the mice was recorded for 10 days (n = 10). * *p* < 0.05, ** *p* < 0.01.

A mouse model was employed for the vaginal colonization assay. Two weeks after the last immunization, mice were intraperitoneally injected with β-estradiol to synchronize the mice into pre-estrus, imitating a condition for GBS colonization in humans. Then, 24 h later, mice were vaginally challenged with GBS serotype Ib. Colony-forming units (CFUs) in vaginal lavage fluid were determined over 35 days following the challenge. CFUs were lower in GBSV6-immunized mice compared to CpG control mice at all detection time points (Figure 5A). By 35 days after challenge, the bacteria were completely cleared in all GBSV6-immunized mice, while up to 10^3^ CFUs were detected in CpG control mice. Similar results were also observed in mice challenged with GBS serotype III (a) (Figure 5B).

To evaluate the protective role of GBSV6 against systemic infections, GBSV6-immunized mice were intravenously challenged with a lethal dose of GBS serotype Ib and lethality was monitored over 10 days. Here, 70% of CpG control mice died on day 1 post-challenge and the lethality increased to 90% within 2 days. In contrast, only 20% of GBSV6-immunized mice died on day 1 and no others died thereafter (Figure 5C). This survival assay was also performed with GBS serotype III (a) challenge. Similar to the results regarding protection against systemic GBS serotype Ib infection, 10% of CpG control mice survived, while 70% of GBSV6-immunized mice survived (Figure 5D). These results indicate that i.n. GBSV6 immunization induces immunity in the reproductive tract and circulation, and it offers cross-protection against GBS serotypes both locally and systemically.

### 3.7. Maternal i.n. GBSV6 Immunization Protected Neonatal Mice against GBS Challenge as Efficiently as Maternal i.m. (Intramuscular) GBSV6 Immunization

We first evaluated the protective effects of GBSV6 in newborns of i.m. immunized dams. Female mice were mated three days following the last i.m. GBSV6/aluminum (Alum) immunization or Alum-only inoculation. Their offspring were challenged with GBS serotype Ib (1 × 10^6^ CFUs/mouse). Less than 7.4% (7/95) of the newborns of the Alum control dams survived, whereas up to 79% (83 of 105) the newborns of the i.m. immunized dams survived (Table 1). This indicates that systemic maternal anti-GBSV6 immunity protects newborns against GBS infection through placental transfer to the fetus. As i.n. GBSV6 immunization induced high levels of serum GBS-specific antibodies, we expected protection of newborns of i.n. immunized dams. Similar to the newborns of i.m. immunized dams, after challenge with the same dose of GBS serotype Ib, up to 85% (91/107) of the newborns of i.n. immunized dams survived compared to 8.3% (9/108) of the newborns of CpG control dams (Table 1). The newborns from i.n. immunized dams were also challenged with GBS serotype III (a) to assess cross-serotype protection. There were significantly more surviving newborns of GBSV6-immunized dams compared to CpG control dams, indicating that the newborns acquired protective levels of maternal antibodies from i.n. immunized dams.

### 3.8. GBSV6 Promoted Clearance of Ascending GBS Infection When Mice Were Immunized through the i.n. but Not the i.m. Route

**Figure 6:**
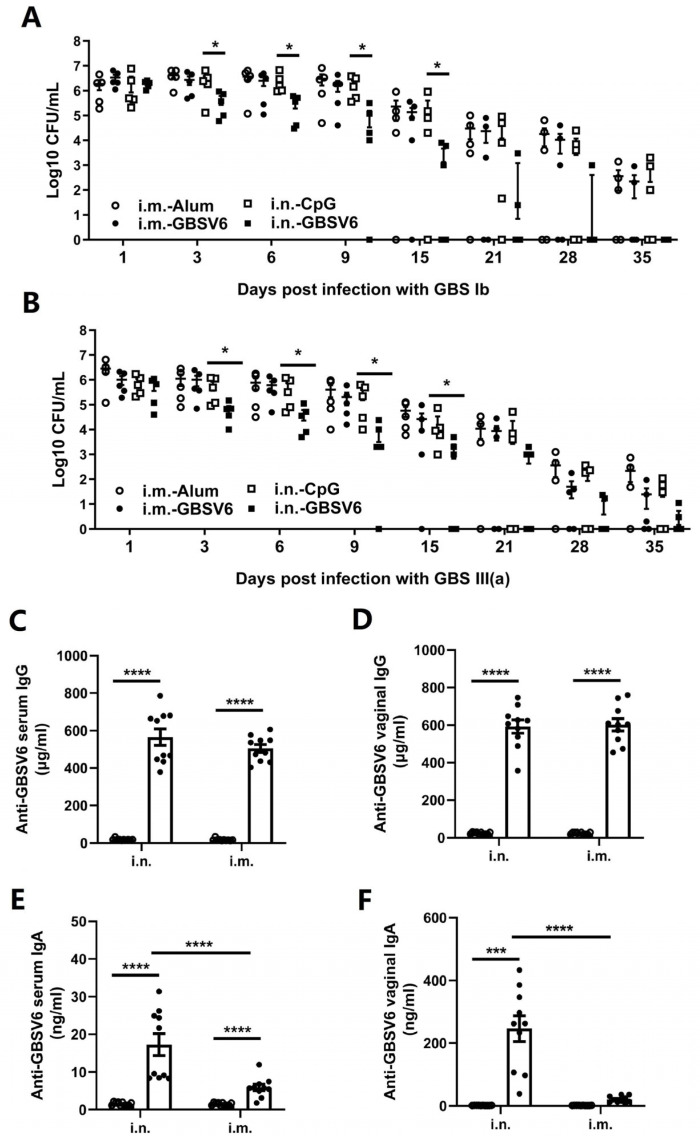
GBSV6 promoted clearance of ascending GBS infection when mice were immunized though the i.n but not the i.m. route. Mice were i.n. immunized with GBSV6/CpG or i.m. with GBSV6/Alum 3 times at 1-week intervals. Control groups were inoculated i.n. with CpG or i.m. with Alum. Two weeks after the last immunization, the mice were intravaginally challenged with GBS serotype (**A**) Ib or (**B**) III (a) and CFUs in the vaginal lavage fluid were determined at the indicated time points after challenge. Data represent mean ± SEM (n = 5). Two weeks after the last immunization, GBSV6-specific (**C**) serum IgG, (**D**) vaginal IgG, (**E**) serum IgA, and (**F**) vaginal IgA antibodies in serum and vaginal lavage fluid were measured by the ELISA. Data are from two independent experiments (n = 10). * *p* < 0.05, *** *p* < 0.001, **** *p* < 0.0001.

In addition to the high levels of serum IgG antibodies, the i.n. immunization induced GBSV6-specific vaginal IgA (Figure 1B,D). This represents an advantage over i.m. immunization, as vaginal IgA offers efficient protection against GBS colonization of the vagina. Mice were immunized with GBSV6 through the i.n. or i.m. route, and bacterial colonization of the vagina was compared after intravaginal challenge with GBS serotype Ib, as before. After the challenge, CFUs were significantly reduced on days 3, 6, 9, and 15 in the i.n. group but not in the i.m. group, while there were no significant CFU reductions in the i.m. group compared to the Alum control group at any detection time point (Figure 6A). Of the 5 mice in the i.n. group, 2 had no CFUs by day 21, while up to 104 CFUs were detected in the i.m. mice. By 35 days following the challenge, no CFUs were found in any mice in the i.n. group, while 2 of the 5 mice in the i.m. group still had CFUs. Similar results were also observed after challenge with GBS serotype III (a) in the i.n. and i.m. groups (Figure 6B). These results indicate that vaginal GBS is cleared faster in the i.n. group than the i.m. group.

Antibodies in the serum and vaginal lavage fluid were compared between the i.n. and i.m. groups. The levels of GBSV6-specific IgG in the serum and vaginal lavage fluid samples were similar between the two groups (Figure 6C,D). Serum IgA in the i.m. group was induced but much lower compared to the i.n. group (Figure 6E). A robust IgA response in the vagina was induced in the i.n. group but was undetectable in the i.m. group (Figure 6F). These observations indicate that the vaginal IgA response induced by immunization through the i.n. route is required for efficient protection against GBS colonization of the reproductive tract.

### 3.9. GBSV6 Induced Long-Lasting Plasma Cells and Th17 Cells and Provided Efficient Long-Term Protection

**Figure 7:**
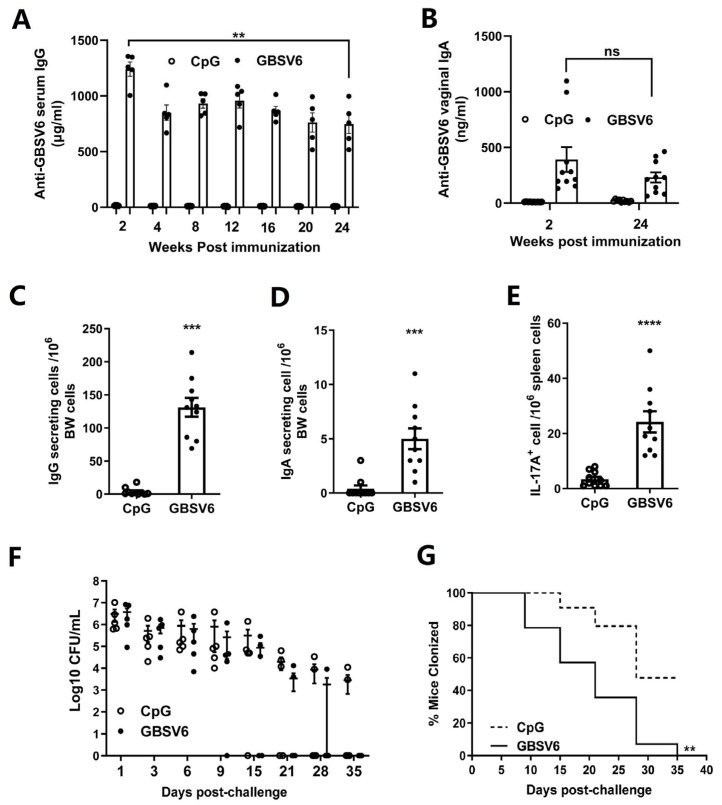
GBSV6 induced long-lasting plasma cells and Th17 cells and provided efficient long-term protection. Six months after the last immunization, GBSV6-specific antibodies were determined by the ELISA, GBSV6-specific antibody-secreting B cells were determined by B cell ELISpot, and IL-17A-secreting T cells were determined by T cell ELISpot. (**A**) Serum IgG, (**B**) vaginal IgA, (**C**) IgG- and (**D**) IgA-secreting B cells in the bone marrow, and (**E**) IL-17A-secreting T cells in the spleen. Data represent mean ± SEM (n = 10). Mice were intravaginally challenged with GBS serotype Ib (1 × 10^7^) and (**F**) CFUs in the vaginal lavage fluid were determined at the indicated time points. (**G**) Percentages of GBS-colonized mice were calculated. Data represent the percentage of mice colonized with GBS. ** *p* < 0.01, *** *p* < 0.001, **** *p* < 0.0001.

To determine whether i.n. immunization can induce long-lasting immunity, antibody and T cell responses were examined six months after the last immunization. The ELISA revealed that i.n. immunized mice maintained 700 ng/mL of serum IgG, although the level was lower compared to the level at 2 weeks after immunization (Figure 7A). Compared to 1 ng/mL in the CpG control group, the vaginal IgA level in i.n. immunized mice at 6 months was 246 ng/mL (range, 38–432 ng/mL), which was comparable to the level at 2 weeks after immunization (Figure 7B).

Consistently, ELISpot showed that the bone marrow levels of IgA^+^ and IgG^+^ long-lasting plasma cells were substantially increased after i.n. immunization compared to CpG control mice (Figure 7C,D). Furthermore, long-lasting IL-17A^+^ T cells that responded to GBSV6 were detected in the spleens of these immunized mice (Figure 7E). These results indicate that i.n. GBSV6 immunization induces long-term immunity.

To assess the efficacy of the long-term immunity, mice were i.n. challenged with GBS serotype Ib at six months after the last immunization, and CFUs in the vaginal lavage fluid were determined. Vaginal CFUs were non-significantly lower in the immunized mice than in control mice (Figure 7F), and by 35 days after the challenge, there were no CFUs in the immunized mice, whereas 50% of the control mice still had CFUs (Figure 7G). This indicates that GBSV6-induced long-lasting immunity promotes faster elimination of GBS colonizing the vagina.

## 4. Discussion

GBS is a leading cause of life-threatening neonatal infections. The diversity of the serotypes of clinical isolates necessitates GBS vaccines that confer cross-serotype protection against the pathogen. While no licensed vaccine exists, conserved GBS proteins are being used for GBS vaccine development for this purpose. Our results showed that i.n. GBSV6 immunization promoted GBS clearance from the vagina and maternal i.n. GBSV6 immunization reduced the death of newborn mice in a serotype-independent manner. Further, immunization induced long-term specific antibody and T cell responses and provided long-term resistance to intravaginal challenge.

We showed that the death of newborns of immunized dams was reduced (compared to controls) to a similar extent in both the i.m. and i.n. groups, but the increase of vaginal IgA antibody and reduction of GBS vaginal colonization were only in the i.n. group and not the i.m. group, indicating that local mucosal IgA significantly contributes to reduced GBS in the vagina. GBS colonization of the reproductive tract is the primary risk factor for EOD during delivery [47], and vaginal mucosal immunity may be critical to inhibit GBS colonization of the reproductive tract and reduce the risk of infection for the newborn. However, we noticed that the reduced vaginal colonization after i.n. immunization seemed not to further reduce the death of newborns compared to i.m. immunization. The impact of the decrease in vaginal CFUs on newborn infection needs to be explored. As GBS ascending to the uterus from the lower genital tract of pregnant women can cause intrauterine fetal death (IUFD), and as parenteral immunization reduces the rate of IUFD [10], it is important to ascertain whether maternal i.n. immunization further reduces this rate. In summary, establishment of immunity in newborns and reduction of bacterial colonization of the vagina of mothers are two promising strategies for effectively constraining GBS diseases. Our results suggest that i.n. GBSV6 immunization induces maternal antibodies, which establish immunity against GBS in newborns for the prevention of newborn death. Additionally, mucosal immunity in the reproductive tract may reduce the risk of GBS transmission from the mother to the baby during pregnancy or at birth [48].

Mice are not a natural host of GBS and are not sensitive to vaginal infection. β-Estradiol increases the sensitivity of mice to vaginal infection and may have some unknown effects that affect the correctness of the interpretation of the results. Verifying the results with different animal models is needed and will be conducted in the future.

The persistence of immunity provides long-lasting protection against reinfection. It has been reported that mucosal immunization induces specific long-term immunity in the genital tract and provides longer lasting protection against intravaginal challenge when compared to systemic immunization with the same antigen. Mucosal compared to systemic immunization is important for the induction of long-term maintenance of immunity in the reproductive tract [49]. We demonstrated that i.n. GBSV6 immunization induced long-lasting immunity against GBS, although the long-term serum IgG and vaginal IgA levels were 60% and 59%, respectively, of the early levels. This long-term immunity would maximize protection for fetus development and against infant infection [4]. Adjuvants used to enhance the immunogenicity of subunit vaccines would induce potential pro-inflammatory reactions, which have a risk of adverse reproductive, teratogenic, or fetal developmental effects [50]. The long immunity would also allow administration of a vaccine prior to pregnancy to reduce the possible risks of vaccination to fetus development [51].

We noticed that the mouse vaginal antibody response to GBSV6 is predominantly IgG. Human studies have shown that in sharp contrast to other mucosal sites, in which secretory IgA represents the dominant Ig isotype, human semen, cervicovaginal secretions, and urine contain higher levels of IgG than IgA [52] due to the minimal associated local lymphoid tissue in the FRT compared to other mucosal sites [53]. However, vaginal IgG, but not IgA, significantly reduced six months after GBSV6 immunization, indicating that IgA is more sustainable than IgG in the vagina.

GBSV6 contains most essential virulence factors of GBS and provides efficient protection. However, clinical GBS isolates in various countries or regions may carry different combinations of virulence factors. GBSV6 can be adjusted according to the prevalence of virulence factors in epidemic GBS strains for better targeting if necessary. Protein-based vaccines provide convenience for the technical adjustment. On the other hand, GBSV6 can be refined by minimizing virulence factors without affecting optimal protection.

CD4^+^ T helper cells (Th) play a central role in immune protection by providing help to B cells and releasing cytokines to protect against pathogenic microorganisms [54]. Th17 cells are critical for protection against bacterial infection in mucosal sites [55]. We previously reported that Th17 cells and the IL-17A that they release are required for efficient clearance of group A streptococcus infections [28]. In addition, IL-17A is associated with the eventual clearance of GBS [56]. We found that immunized mice developed a strong Th17 response in the spleen and FRTs, suggesting that the Th17 response plays an important role in clearing GBS.

The presence of non-circulating, tissue-resident memory T cells (Trms) in the desired tissues is important for long-term vaccine-induced immunity in these tissues, which reduces the incidence of infection [57,58]. However, we did not find these cells in the vaginal tissue, though GBSV6-specific memory T cells were detected in the spleen. Evidence indicates that Trms preferentially populate the site of immunization [59,60]. Thus, efficient generation of Trms in the FRT requires intravaginal stimulation. Whether combined i.n. and intravaginal immunization can strengthen the long-lasting protective immunity in the vagina will be tested in our future study.

## 5. Conclusions

In this study, we demonstrated that immunization with a set of six conserved virulence factors involved in GBS pathogenesis through the i.n. route induces serotype-independent immunity both systemically and in the vagina, and thus provides protection against GBS. The immunity is long-lasting and reduces the death of newborn mice and GBS colonization of the vagina. The results suggest that multiple conserved virulence factors and the i.n. route can be considered as strategies for advancing GBS vaccine development.

## Figures and Tables

**Table 1:** GBSV6 protected neonatal mice against GBS challenge.

Immunization Route, Vaccine	GBS Strains	No. of Mice Protected /No. of Mice Treated	Protection Ratio
Intranasal CpG	GBS Ib	9/108	
GBSV6	GBS Ib	91/107	83.7 *
CpG	GBS III (a)	15/101	
GBSV6	GBS III (a)	77/98	74.8 *
Intramuscular Alum	GBS Ib	7/95	
GBSV6	GBS Ib	83/105	77.4 *

* Female mice were intranasally (i.n.) immunized with GBSV6/CpG or i.m. with GBSV6/Alum, as described in Figure 1. Control mice were inoculated with CpG or Alum alone. Mice were mated 3 days after the last immunization. The offspring were intraperitoneally challenged with GBS serotype Ib or III (a) (2 × 10^6^) within 48 h of birth. Survival of neonatal mice was monitored for 3 days after challenge. Protection values were calculated as follows: [(% dead in control group-% dead in vaccine group)/% dead in control group] × 100. Data are from two independent experiments. * *p* < 0.05 (Fisher’s exact test).

## Data Availability

All data that this study is based upon are available from the corresponding author upon request.

## Supplementary Materials

The following supporting information can be downloaded at: https://www.mdpi.com/article/10.3390/vaccines11091459/s1, Figure S1: Purified recombinant proteins of GBSV6. Purified recombinant proteins were separated by 12% sodium dodecyl sulfate-polyacrylamide gel electrophoresis and stained with 0.25% Coomassie brilliant blue. Lane 1, molecular weight marker (MW); lane 2, SrtA; lane 3, ScpB; lane 4, CspA; lane 5, Srr2; lane 6, BibA; lane 7, FbsB. Table S1: Primers Used for Cloning GBSV6 genes. Underlined nucleotides denote enzyme restriction sites.
