# Supplementary material for: Immunization with Multiple Virulence Factors Provides Maternal and Neonatal Protection against Group B Streptococcus Serotypes
Source: Vaccines (Basel). 2023 Sep 5;11(9):1459. doi: 10.3390/vaccines11091459 (PMC10535937; doi:10.3390/vaccines11091459)
Supplement: Supplementary file 1 [file vaccines-11-01459-s001.zip › vaccines-2565411-supplementary.pdf]

Supplementary material

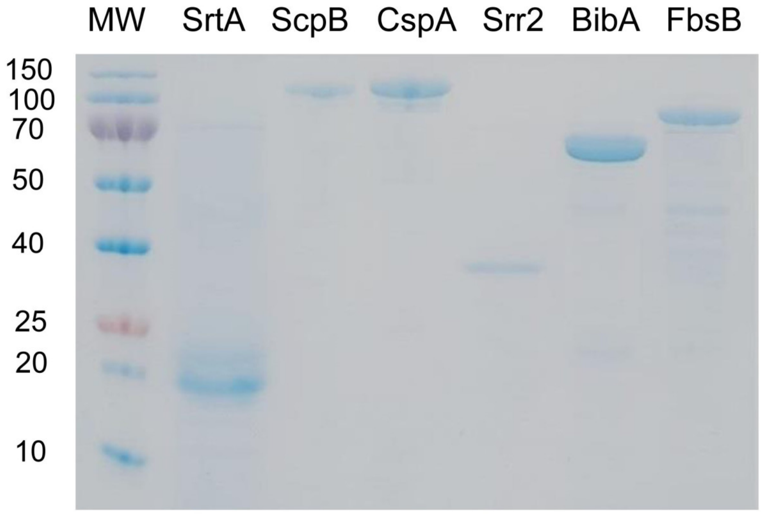

Figure S1. Purified recombinant proteins of GBSV6. Purified recombinant proteins were separated by 12% sodium dodecyl sulfate-polyacrylamide gel electrophoresis and stained with 0.25% Coomassie brilliant blue. Lane 1, molecular weight marker (MW); lane 2, SrtA; lane 3, ScpB; lane 4, CspA; lane 5, Srr2; lane 6, BibA; lane 7, FbsB.

Table S1 Primers Used for Cloning GBSV6 genes. Underlined nucleotides denote enzyme restriction sites.

Gene name	Primer（5'-3'）
SrtA Forward	CATGCCATGGGCTCTGCTCAAACGAAATCACA
SrtA Reverse	CCGCTCGAGGAGATTAATTTGATTATATT
ScpB Forward	ATGACCATGGGCAATACTGTGACAGAAGACACTCC
ScpB Reverse	CCGCTCGAGAGAGTGGCCCTCCAATAGCTT
CspA Forward	CATGCCATGGGCGATTCTGTCATAAATAAGCC
CspA Reverse	CCGCTCGAGATTGCCAATATTGATCAAATCT
BibA Forward	CGAGCTCCACGCGGATACTAGTTCAGGA
BibA Forward	ACGCGTCGACACCTCTGGTAAGGTCTTGAA
Srr2 Forward	CATGCCATGGGCTCAGAAGCGGCAACGACCGCTAGAG
Srr2 Reverse	AAAGGATCCAGCATTTACATCTGAATA
FbsB Forward	CATGCCATGGCCGGGATAACTAAAG
FbsB Forward	CCGCTCGAGCTCTTTTATACGCGATGAG
